# Developing information material to support the assessment of palliative care needs in dementia: a qualitative participatory approach

**DOI:** 10.1186/s12904-026-02066-4

**Published:** 2026-03-13

**Authors:** Daniela Gesell, Julia Wikert, Katerina Hriskova, Arianita Veliqi, Claudia Bausewein

**Affiliations:** Department of Palliative Medicine, LMU University Hospital, LMU Munich, Munich, Germany

**Keywords:** Dementia, End-of-life care, Palliative care, Nursing home, Long-term care

## Abstract

**Background:**

There are approximately 1.8 million people living with dementia in Germany, of whom many present with a wide range of symptoms and needs. The assessment of these symptoms is often challenging for nursing staff in long-term care settings. The dementia-specific version of the Integrated Palliative Outcome Scale (IPOS-Dem) can support this assessment by enabling the timely identification of the needs of people living with dementia. To facilitate the use of IPOS-Dem, a user-friendly manual is required. The aim of this study is therefore to develop information material for assessing palliative care needs among people living with dementia through an iterative and participatory process tailored to professional caregivers, and to explore the content requirements of these materials from the caregivers’ perspective.

**Methods:**

A multi-stage qualitative study involving a Patient and Public Involvement (PPI) group and a focus group with professionals was conducted. The manual was developed through an iterative and participatory process, which included revision based on input from the PPI group and the validation of the final version in a structured focus group discussion. The data was analyzed using Mayring’s qualitative content analysis.

**Results:**

The PPI group consisted of three, the focus group of seven people (median age = 51 years; median work experience = 29 years). Feedback from the discussions encompassed themes that were incorporated into the revised version of the manual, including reducing continuous text, adding graphical elements to improve readability, revising the layout, and refining the case example to describe observable differences in a more concrete way. Linguistic revisions, such as simplifying and shortening phrasing, as well as making the manual more engaging and motivating, were also implemented. The manual was rated by the participating professionals as helpful and practice-oriented, supporting the systematic assessment of palliative care needs.

**Conclusions:**

The participatory and iterative development process ensured that the manual reflects the practical needs and perspectives of professional caregivers, thereby enhancing its relevance and acceptance in dementia care.

**Supplementary Information:**

The online version contains supplementary material available at 10.1186/s12904-026-02066-4.

## Background

In Germany, an estimated 1.8 million people are living with dementia, with around 900 new cases diagnosed every day. By the year 2050, the German Alzheimer’s Association expects the number of people affected to rise to up to 2.7 million [1]. More than two-thirds of people living with dementia reside in private households, while about one-fifth live in nursing and long-term care settings [2]. People affected often suffer from a wide range of symptoms and problems, such as shortness of breath, pain, or difficulties with eating and drinking [3]. It has been shown that many of those affected develop psychiatric disorders over the course of the disease, such as anxiety, depression, hallucinations, or delusions [4]. Targeted interventions can reduce behavioral symptoms in people living with dementia and improve their quality of life [5]. A major challenge in the care of people living with advanced dementia is their often severely reduced ability to communicate, which can lead to important needs or symptoms being unrecognized [6]. Comprehensive symptom assessment is essential to identify emerging problems and needs at an early stage and to ensure timely medical and nursing interventions, thereby reducing avoidable distress.

To enable the timely identification of these needs, the *Integrated Palliative Care Outcome Scale for Dementia* (IPOS-Dem) was developed at King’s College London. The IPOS-Dem is designed to support the routine assessment of care home residents living with dementia, taking into account the common symptoms and issues, e.g. the ability to interact positively or concerns about anxiety experienced by this group [7]. The questionnaire is a dementia-specific adaptation of the *Integrated Palliative Care Outcome Scale (IPOS)*, which has been tested and applied in numerous clinical settings and among various patient groups [8]. Since people living with advanced dementia are often unable to communicate their needs verbally, the IPOS-Dem questionnaire is completed based on assessments made by professional caregivers and/or family members or close relatives.

Findings and results from a cognitive interview study on the cultural adaptation of the IPOS-Dem indicated that the development of training materials is necessary to make the questionnaire accessible to users within the specific cultural context. This is essential to enable the successful implementation of an IPOS-Dem-based palliative care assessment by healthcare professionals and other caregivers [9]. Participatory approaches are well established in the development and implementation of nursing care tools, in particular, focus groups are used to identify additional relevant themes and to refine the wording of the materials, thereby ensuring that they are closely aligned with the practical needs of future users [10]. Therefore, the primary aim of this study is to develop information material for assessing palliative care needs among people living with dementia through an iterative and participatory process tailored to professional caregivers, and to explore the content requirements of these materials from the caregivers’ perspective.

## Methods

### Study design

**Fig. 1:**
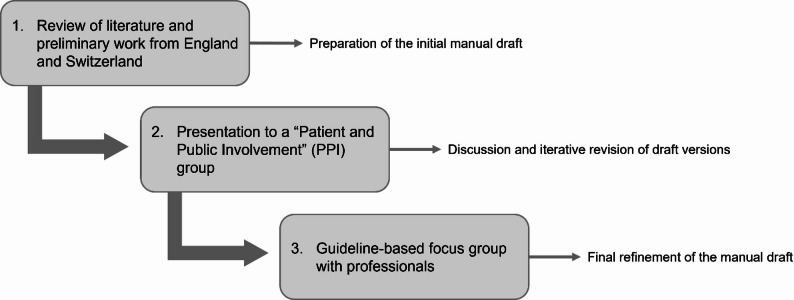
Flowchart of the study design

The information material for the use of IPOS-Dem were developed within a multi-stage qualitative study design incorporating participatory elements (see Fig. 1). The study was developed and reported in accordance with the Consolidated Criteria for Reporting Qualitative Research (COREQ) checklist [11]. This ensured comprehensive documentation of the research team, study design, data collection and analysis, as well as reflexivity and participant involvement throughout the multi-stage, participatory qualitative process. The Guidance for Reporting Involvement of Patients and the Public (GRIPP2) short form checklist was also used to enhance transparency of patient and public involvement [12]. 

Participatory processes are well established in the development and implementation of nursing care tools. Focus groups are a proven method for incorporating and evaluating practical feedback from nursing professionals. The group dynamic makes it possible to discuss and explore interesting aspects in more detail [13, 14]. 

### Data base and preliminary work

The development of the information material for the use of the IPOS-Dem was based on existing resources from various contexts: a manual developed at King’s College London, training materials on the IPOS from the Department of Palliative Medicine at LMU University Hospital [15], and training documents from an implementation program in Switzerland. The contents of the different manuals were compared and evaluated using a comparative matrix. In addition, a qualitative supplementary analysis [16] was conducted based on fully anonymized transcripts of cognitive interviews that had previously been collected as part of the IPOS-Dem validation process at the Department of Palliative Medicine at LMU University Hospital [9]. The aim was to identify additional thematic areas relevant to the revision of the materials. The evaluated resources were then compiled into a preliminary manual.

### Participants and data collection

The manual was developed using an iterative, participatory approach. An initial version of the manual was discussed in a *Patient and Public Involvement* (PPI) group established in person in the Department of Palliative Medicine at LMU University Hospital, comprising family caregivers and people with lived experience of palliative care. The aim of the PPI group was to inform the preliminary development and refinement of the manual. Feedback from this PPI group was documented in written field notes and used to revise and refine the manual. The PPI discussion was not audio-recorded and did not constitute formal qualitative data collection. Subsequently, the revised manual was discussed in one audio-recorded online focus group comprising professional caregivers from nursing homes of people living with dementia and experts in the field of dementia, which formed the basis of the qualitative analysis.

As part of a broader research programme on person-centred outcome measures in nursing homes, a quantitative study using the IPOS-Dem questionnaire was conducted in multiple facilities in Bavaria. For the present focus group study, nursing staff were recruited from four of these participating nursing homes. Eligible staff members who were already familiar with the IPOS-Dem questionnaire were invited to participate. Participation was voluntary. In addition, two participants were recruited from the Christophorus Hospice Association Munich (CHV), a palliative care organization, to incorporate further expertise in end-of-life care for people living with dementia. The project team developed a literature-based discussion guide, designed to partially structure the discussion and ensure data comparability (see Supplementary Material 1). The guide followed the four-step process described by Helferich (collecting, reviewing, sorting, subsuming) [17] and was designed to maintain openness and flexibility, allowing the moderator to respond to the dynamics of the discussion. Participants received the most recent version of the preliminary information material in advance for preparation.

The 75-minute focus group was moderated by a project team member (JW, female research associate with a doctoral degree and extensive experience in qualitative research), and supported by a PowerPoint presentation of the preliminary manual (content and structure). The group consisted of seven participants, allowing for active exchange while ensuring the inclusion of all members. It was documented whether any participants were known to the research team from previous professional interactions. No prior relationships were established specifically for the purpose of the study. Participants were informed about the researchers’ professional background and the study aims. According to the researchers’ assessment, such prior contact was not expected to influence participants’ responses.

With the participants’ consent, the discussions were audio-recorded and transcribed verbatim in compliance with data protection regulations. In addition, a second project team member (DG) was present as a non-participating observer and took non-personal field notes during the focus group. Standardized demographic information of the participants was collected in written form via questionnaire.

### Data analysis

The collected field notes from the PPI group and the verbatim transcripts from the focus group were analyzed using a structured content analysis approach [18]. As the focus group was conducted and transcribed in German, data analysis was performed using the original German transcripts. Coding and analysis were conducted manually without the use of qualitative data analysis software. First, theory-driven main categories were established based on the research group’s previous work and the results from the PPI group. Coding and category development were conducted by one researcher (DG) and discussed within the research team to refine the category system. Subsequently, an inductive category system was developed in which text segments were assigned to main and subcategories. The deductive category formation was guided by theoretical assumptions, while subcategories were derived inductively from the material. Relevant text passages were highlighted, paraphrased, and organized by meaning within each category. In the next step, the paraphrases were condensed into category-specific key statements. The focus was not on the frequency of individual aspects but rather on the relevance of aspects related to the content, structure, and applicability of the manual to the research question. Key themes identified were discussed iteratively within the project team (JW, DG, KH and CB). Based on these discussions, successive revisions of the manual were prepared by one project team member (DG) to ensure a reflective analytical process, the intersubjective traceability of the results and that they met the practical needs of the target users. The insights gained were gradually incorporated into revisions of the manual. The revised version of the manual was not returned to focus group participants or the PPI group for further feedback.

For the preparation of the manuscript, relevant quotations and category labels were translated into English by a member of the research team (DG) and cross-checked within the research team to preserve meaning and contextual nuances. The manual was developed in German, as the participating professionals were working in German long-term care settings.

## Results

A total of three members of the PPI group participated in a discussion on the version of the manual, after which a focus group consisting of seven participants was conducted. Details of the participants are presented in Table 1.

**Table 1:** Sociodemographic characteristics of the PPI and focus groups

	PPI group	Focus group
Number of participants	3	7
gender	2 male, 1 female	7 female
Age (years)	Median 64 (Range 62–70)	Median 51 (Range 41–65)
Profession		3 Registered Nurses, 4 Others (social worker, social pedagogue, occupational therapist, care home manager)
Professional experience (years)		Median 29 (Range 15–35)
Setting	LMU University Hospital, 60 min	Online, 75 min

### Results from the PPI group

The feedback from the PPI group discussion resulted in five main themes: (1) structure and layout, (2) readability and language, (3) use of graphical elements, (4) clarity and concreteness of case examples, and (5) motivational design. These themes were incorporated into a revised version of the manual. While some thematic overlap with the subsequent focus group analysis was observed, the PPI themes primarily addressed presentation, readability, and usability aspects of the manual. In detail, participants suggested reducing continuous text, adding graphical elements to enhance readability, and revising the layout. Furthermore, formulating the case example in a more concrete way and describing observable differences. In addition to linguistic revisions, such as using simpler and shorter phrasing, participants also suggested designing the manual in a more engaging and motivating way.

### Results from the focus group

The focus group analysis resulted in six main themes for improving the manual: (1) shortening and simplifying text, (2) strengthening the focus on the needs of people living with dementia and promoting a perspective shift, (3) presenting the benefits of IPOS-Dem clearly and early in the manual, (4) revising the case example to make it less abstract and more practice-oriented, (5) optimizing layout and structure, and (6) ensuring relevance to everyday nursing practice.

In detail, participants emphasized that the current version of the manual was too complex and text-heavy. Long sentences and extensive continuous text made reading difficult and led to important content being easily overlooked. To enhance comprehensibility, participants recommended shortening and simplifying the text. Clear and concise wording should help to ensure that key information can be more quickly understood and applied in everyday care practice.

*“In the world where there are so many letters*,* where so much has to be documented*,* where so much has to be done… it should be something that clearly and briefly catches the eye.” (P_3)*.

*‘Where there are many words*,* much goes unread.’ (P_3)*.

Another key aspect was the stronger focus on the needs and perspective shift required from caregivers. The emphasis should not be on what caregivers ‘have to do,’ but on what people living with dementia actually need. The caregivers’ task is to observe and identify these needs. Participants in the focus group emphasized that the manual should support this shift in perspective and highlight the importance of needs-oriented care.

*‘So that it is emphasized once again: it’s not about what we have to do*,* but rather that the resident dictates what we should do - observing and recognizing their needs.’ (P_3)*.

The advantages of the IPOS-Dem should also be clearly and explicitly communicated early on. Participants emphasized that the practical benefits of the IPOS-Dem need to be presented more prominently and at the very beginning. They particularly highlighted that the instrument helps to quickly and systematically derive actions for the care team. At the same time, it was noted that the benefits become apparent only if the insights gained are promptly integrated into care practice. The manual should therefore place greater emphasis on the specific added value that the use of the instrument offers in everyday care.

*‘I would benefit from it*,* I can quickly and systematically derive actions for the team from it. That would really be a great benefit for me.’ (P_4)*.

*‘I’m missing a bit […] that the insights are promptly incorporated into care. I benefit from completing this care form only if I can quickly see the advantages. Otherwise*,* it won’t be attractive for caregivers.’ (P_4)*.

Participants also stated that the case example used in the manual was too abstract and therefore difficult to relate to. The example should be concrete and rooted in everyday practice, creating an immediate and clear mental image. Realistic, practice-oriented situations help to improve understanding of the content and support its applicability in daily care.

*‘For example*,* to say*,* a distorted facial expression due to pain. Or the second resident keeps walking around all the time*,* even though he is in pain. So really*,* just provide examples that can serve as a basis for interpretation.’ (P_4)*.

*‘What I would find important is that an image emerges […] so that when someone reads it*,* they immediately have a picture in mind.’ (P_1)*.

*‘Maybe something really simple*,* like groaning during a transfer or when getting up […] something that’s familiar from everyday life and easy to grasp.’ (P_2)*.

Participants also noted that the manual requires a clearer layout and a more organized structure. They emphasized the importance of highlighting key aspects, such as the needs of people living with dementia. In addition, the core messages of individual sections should be visually emphasized, for example through bold text. This would make important content stand out more quickly and facilitate its application in everyday practice.

*‘Certain aspects should really be highlighted specifically*,* for example*,* the needs.’ (P_4)*.

*‘I basically had the idea of whether it would be possible to visually highlight the key message of each paragraph*,* i.e. make it bold*,* […] so that the most important points stand out.’ (P_2)*.

Eventually, a key consideration highlighted was the seamless integration of IPOS-Dem into routine nursing care. It was recommended that clear guidance be provided on the optimal use case for IPOS-Dem implementation as well as the designation of responsible profession(s) to ensure effective integration. Case discussions were identified as a suitable setting for this, as they are often initiated when a patient’s condition deteriorates or when the end of life is foreseeable. This would ensure that the questionnaire is used practically and meaningfully integrated into existing processes.

*‘Where can it best be integrated*,* and by whom? Who would then be responsible?’ (P_1)*.

*‘Where would be the best place for that? I could imagine perhaps during case discussions*,* because we […] hold case discussions specifically when we feel that someone is not doing well*,* when they are at the end of their life.’ (P_1)*.

### Contents of the final version of the manual

The final version of the manual comprises twelve pages. It includes a foreword to the IPOS-Dem questionnaire and a general introduction to dementia at the end of life. The manual emphasizes the unique needs of individuals living with dementia, prioritizing the preservation of their well-being and quality of life, especially during the end-of-life phase. Additionally, a comprehensive overview of the IPOS-Dem questionnaire is provided, highlighting its components and describing the benefits of its application within care settings. A specific case study is used to demonstrate its application in everyday nursing care. An additional component addresses the evaluation of IPOS-Dem, illustrating the significance of elevated scores within nursing care and highlighting critical areas that require focused attention. As a key result of the focus group discussion, recommendations on using the IPOS-Dem in everyday nursing care have been added. The manual concludes with a general information box on standardized questionnaires and outcome measurement in the care of individuals with health conditions (see Supplementary Material 2).

## Discussion

The manual developed is a low-threshold tool designed to support needs-based care for people living with dementia. The close involvement of professional caregivers enabled practical content to be integrated into the manual. Feedback from the PPI and focus group indicated that a manual for using the IPOS-Dem questionnaire is widely regarded as useful and highlighted the importance of clear language, practical relevance, and integration into everyday nursing routines (e.g. case discussions). It was seen as essential to emphasize the added value of IPOS-Dem for caregivers for acceptance and sustained use. Regular use of the IPOS-Dem questionnaire represents a change to everyday nursing practice and requires attention to contextual factors such as resource and knowledge availability [19]. 

Our findings contribute to the discussion on how manuals and training materials for assessment tools should be designed in long-term care settings. Previous research has mainly focused on the implementation of such tools, particularly in the context knowledge translation processes [20, 21], whereas less attention has been given to the structure and presentation of the manuals themselves. Our results suggest that usability depends not only on content accuracy but also on how well manuals reflect everyday workflows and practical realities in long-term care. In this regard, our study contributes to a more practice-oriented understanding of manual design. A recent review on training healthcare professionals in assessing the health needs of older adults similarly emphasizes structured educational materials and alignment with everyday clinical workflows [22]. Our findings extend this perspective by providing practical insights from long-term care professionals on how manuals can be adapted to nursing home contexts.

Beyond the structural aspects of the manual design, the content-related focus of the manual was also shaped by the participants’ emphasis on person-centred care. The results highlighted the need for a stronger focus on the individual needs of people living with dementia. In particular, participants emphasized the importance of promoting a perspective shift towards person-centered care, adapting communication to cognitive limitations, and addressing everyday nursing realities. These aspects were incorporated into the final manual through strengthened person-centered guidance, a revised more practice-oriented case example, and practical recommendations for use in routine care. This aligns with international recommendations on palliative and dementia care, which emphasize person-centered care, communication, the optimal treatment of symptoms and ensuring comfort, among other things [23]. 

Moreover, an important dimension that merits further exploration is the influence of contextual and organizational factors on the successful implementation of the IPOS-Dem manual in routine care. While the participatory development process has enhanced the manual’s relevance, important factors such as staff engagement, contextual working conditions for the sustained use, and institutional and managerial support play critical roles in determining whether such tools are feasibly integrated and sustained over time [24]. 

Finally, further developments are planned to include an adapted version for relatives and translations into additional languages. These extensions aim to facilitate the use of the manual across diverse care settings and to promote collaboration with family members. Furthermore, future evaluations could examine the extent to which the manual influences the implementation of the IPOS-Dem questionnaire and enhances the identification of the needs of people living with dementia. While the present study offers valuable insights for the ongoing refinement of the manual, it does not yet provide empirical evidence regarding the effectiveness of the implemented modifications in practice. Consequently, subsequent research should investigate the manual’s practical applicability in routine nursing care to determine whether the revisions contribute to improving the usability of IPOS-Dem.

### Strengths and limitations

A key strength is the participatory development process, which involved people from various disciplines and backgrounds. Their feedback ensured that the manual closely aligned with everyday nursing practice. This approach also helped identify practical design principles that may be relevant for manuals used in long-term care facilities more broadly. In addition to the strengths, there are also limitations to report. A key limitation is the relatively small number of focus group participants. Although appropriate for qualitative exploration, the findings may primarily reflect the specific contexts of the institutions from which participants were recruited, potentially limiting transferability to other settings. Moreover, while the PPI discussion enhanced the practical relevance of the manual, it was documented through written field notes rather than audio-recorded transcripts, which may have limited the depth and nuance of data capture. Finally, the last version of the manual was not returned to participants for member checking. Although the development process was iterative, the absence of formal validation with participants may have limited the opportunity to confirm whether the revisions fully reflected their perspectives.

## Conclusion

The participatory development process ensured the manual’s relevance and usability for professional caregivers. While the manual was regarded as both helpful and practical, further research is necessary to rigorously assess its effectiveness and feasibility within routine care settings. Planned future adaptations, including translations and a modified version for family caregivers, are expected to enhance its applicability across diverse care settings.

## Supplementary Information

Supplementary Material 1.

Supplementary Material 2.

## Data Availability

The IPOS-Dem questionnaire can be downloaded from the following URL: [https://pos-pal.org/maix/ipos-dem.php](https:/pos-pal.org/maix/ipos-dem.php) [25]. The datasets generated and/or analyzed during the current study are not publicly available but are available from the corresponding author on reasonable request. The final manual will be made publicly accessible via the website of the Department of Palliative Medicine at LMU University Hospital.

## Abbreviations

CHV: Christophorus Hospice Association Munich
IPOS: Integrated Palliative Care Outcome Scale
IPOS-Dem: Integrated Palliative Care Outcome Scale for Dementia
PPI: Patient and Public Involvement
