# Supplementary material for: Developing information material to support the assessment of palliative care needs in dementia: a qualitative participatory approach
Source: BMC Palliat Care. 2026 Mar 13;25:90. doi: 10.1186/s12904-026-02066-4 (PMC13063761; doi:10.1186/s12904-026-02066-4)
Supplement: Supplementary file 1 — Supplementary Material 1. [file 12904_2026_2066_MOESM1_ESM.pdf]

Supplementary Material 1. Discussion guide for focus group with professionals

Timeline	Topic/Question
10 minutes	Welcome & introduction<br>• Procedure & rules (e.g., every opinion counts, there are no wrong answers)<br>• Overview of the manual:<br>What is IPOS-Dem? (purpose, area of application)<br>Why was the manual created?<br>What does it contain (brief overview of the structure: introduction, application steps, tips, etc.)<br>How is it intended to be used? (proxy completion in everyday life by nursing staff)
15 minutes	Introduction: First impressions<br>• What was your first impression of the manual?<br>• What was particularly easy/difficult to understand?
25 minutes	Structure & contents of the manual<br>• Which parts were helpful?<br>• Where was something too long / too short / unclear?<br>• Were there any technical terms that were difficult to understand?
15 minutes	Suggestions for improvement & open questions<br>• What would you change about the manual?<br>• Is there any information missing?
20 minutes	Everyday use<br>• Can you imagine using IPOS-Dem in everyday life?<br>• What would you need to make it practical?<br>• What could help (e.g., training, reminders, short versions, explanatory videos)?
10 minutes	Conclusion & Acknowledgements<br>• Summary of the most important points
