# Supplementary material for: Developing information material to support the assessment of palliative care needs in dementia: a qualitative participatory approach
Source: BMC Palliat Care. 2026 Mar 13;25:90. doi: 10.1186/s12904-026-02066-4 (PMC13063761; doi:10.1186/s12904-026-02066-4)
Supplement: Supplementary file 2 — Supplementary Material 2. [file 12904_2026_2066_MOESM2_ESM.pdf]

Supplementary Material 2. Overview of changes during the development of the manual

Section of the manual	Initial draft	Modifications implemented	Final version
Foreword / Introduction	Introduction to IPOS-Dem questionnaire and dementia at end of life.	Clarify benefits of IPOS-Dem questionnaire early; place stronger emphasis on practical relevance; simplify language.	The foreword and introduction were revised to include an early, accessible explanation of the added value of IPOS-Dem questionnaire for caregivers.
Benefits of the assessment	Overview of benefits based on literature and preliminary work.	Confirmed order of content; shortened overly detailed points; reworded into more practical language; restructured so that benefits are explained after the IPOS-Dem questionnaire.	Benefits are presented after the introduction of the IPOS-Dem questionnaire, using simple language and focusing on essential points only.
IPOS-Dem questionnaire	Explanation of the content and structure of the IPOS-Dem questionnaire.	Integrate sections of the questionnaire; provide a more visual presentation; emphasize the role of nursing staff.	The IPOS-Dem questionnaire is explained in detail and the role of nursing staff is described. Subdomains of the assessment are presented graphically in an information overview.
Specific case study	Abstract and general case description without concrete examples.	Make the case more concrete, describe observable differences, and increase practical relevance.	The case study was rewritten to be more practice oriented, with concrete observable differences described.
Evaluation of IPOS-Dem questionnaire	Explanation of case example with interpretation.	No adjustment necessary.	Explanation of the case example with interpretation was retained without changes.
Recommendations on using the IPOS-Dem questionnaire in everyday nursing care	Not included in the manual.	Add description for use in everyday work; provide assistance with integrating the IPOS-Dem questionnaire.	Detailed assistance for integrating the IPOS-Dem questionnaire into everyday work was added. The information has been presented in graphic form for improved readability and clarity.
Information on outcome measurement	Brief reference to standardized tools.	No adjustment necessary.	A brief reference to standardized outcome measurement tools was retained without changes.
